# Long-term Control of Nasal Tip Position: Quantitative Assessment of Caudal Septal Extension Graft

**DOI:** 10.1093/asjof/ojad027.006

**Published:** 2023-04-14

**Authors:** Vikram Mookerjee, Jinesh Shah, Martin Carney, David Alper, Derek Steinbacher

**Affiliations:** Yale University School of Medicine, New Haven, CT; Yale University School of Medicine, New Haven, CT; Yale University School of Medicine, New Haven, CT; Yale University School of Medicine, New Haven, CT; Yale University School of Medicine, New Haven, CT

## Abstract

**Goals/Purpose:**

Control of nasal tip position is critical in final rhinoplasty outcomes. Two frequent methods of exerting tip control are columellar strut and caudal septal extension grafts. Past work has demonstrated that septal extension grafts are better able to preserve tip rotation over time. However, there is no quantitative data describing long term projection and rotation with septal extension grafts, which is essential to the final aesthetic result. The purpose of this study was to analyze long term maintenance of tip projection and rotation following septal extension graft.

**Methods/Technique:**

A retrospective cohort study of adult patients undergoing open rhinoplasty was conducted. Open rhinoplasty with three-dimensional data and use of septal extension graft were collated. Excluded were closed rhinoplasty, cleft rhinoplasty, tip rhinoplasty only, cases where deprojection or derotation was implemented, and cases less with than 2 years of follow up. All patients underwent osteotomies, tip projection, and tip rotation. Three-dimensional photogrammetric evaluation of nasal tip position was performed preoperatively and postoperatively (Vectra; Canfield Scientific, Parsippany, N.J.). Anthropometric points were analyzed preoperatively, 1 week postoperative, 6 weeks postoperative, 2 years postoperative, and at final documented follow up. Outcome variables were tip projection, tip rotation, and nasal length. Nasal projection was measured from the piriform to pronasale, and nasal length was measured from radix to pronasale. Nasolabial angle was defined using three points (tip, subnasale, and Cupid’s bow). Results were compared statistically using dependent t-test.

**Results/Complications:**

A total of 20 patients were included. Average follow up time was 3.3 ± 1.3 years (2.0 to 6.6 years). 15 patients underwent primary rhinoplasty and 5 underwent secondary rhinoplasty. Overall, 75 percent were female, with an average age of 25.5 years at the time of surgery. From 1 week to 6 weeks postoperative, there was a decrease in rotation (-4.3%, p<0.01) as well as an observed but not statistically significant decrease in projection (-1.5%) and nasal length (-0.9%). There was no statistically significant decrease in projection, rotation, or nasal length from 6 weeks postoperative to 2 years postoperatively, or from 6 weeks postoperatively to final follow up (2.0 to 6.6 years).

**Conclusion:**

Nasal tip projection and rotation appear to decrease from the immediate postoperative position, likely due to resolving edema. In this study, patients that underwent open rhinoplasty with septal extension graft experienced modest loss of projection and rotation until 6 weeks postoperative, but maintained projection and rotation from 6 weeks postoperative to 2 years and beyond. This study provides evidence that septal extension graft maintains long term changes in tip projection and rotation.

